# The effects of smooth pursuit adaptation on the gain of visuomotor transmission in monkeys

**DOI:** 10.3389/fnsys.2013.00119

**Published:** 2013-12-23

**Authors:** Seiji Ono

**Affiliations:** Department of Ophthalmology and Washington National Primate Research Center, University of WashingtonSeattle, WA, USA

**Keywords:** visuomotor, smooth pursuit, adaptation, perturbation, rhesus macaque

## Abstract

Smooth pursuit eye movements are supported by visual-motor systems, where visual motion information is transformed into eye movement commands. Adaptation of the visuomotor systems for smooth pursuit is an important factor to maintain pursuit accuracy and high acuity vision. Short-term adaptation of initial pursuit gain can be produced experimentally using by repeated trials of a step-ramp tracking with two different velocities (double-step paradigm) that step-up (10–30°/s) or step-down (20–5°/s). It is also known that visuomotor gain during smooth pursuit is regulated by a dynamic gain control mechanism by showing that eye velocity evoked by a target perturbation during pursuit increases bidirectionally when ongoing pursuit velocity is higher. However, it remains uncertain how smooth pursuit adaptation alters the gain of visuomotor transmission. Therefore, a single cycle of sinusoidal motion (2.5 Hz, ± 10°/s) was introduced during step-ramp tracking pre- and post-adaptation to determine whether smooth pursuit adaptation affects the perturbation response. The results showed that pursuit adaptation had a significant effect on the perturbation response that was specific to the adapted direction. These results indicate that there might be different visuomotor mechanisms between adaptation and dynamic gain control. Furthermore, smooth pursuit adaptation altered not only the gain of the perturbation response, but also the gain slope (regression curve) at different target velocities (5, 10 and 15°/s). Therefore, pursuit adaptation could affect the dynamic regulation of the visuomotor gain at different pursuit velocities.

## Introduction

Smooth pursuit eye movements allow us to stabilize the image of a moving object on or near the fovea. Smooth pursuit is supported by visuomotor systems, where visual motion information is transformed into motor commands (Krauzlis, 2004; Lisberger, 2010). Pursuit initiation is thought to be driven, in part, by visual motion signals from cortical areas, such as the middle temporal area (MT) and the medial superior temporal area (MST; Newsome et al., 1985, 1988; Dursteler and Wurtz, 1988). The first 100 ms of pursuit tracking is defined as an open-loop response that occurs before the time of the visual feedback, while steady-state pursuit gain is maintained by a feedback system (Robinson et al., 1986; Nuding et al., 2008). As pursuit eye velocity approaches target velocity, visual motion driven MT/MST neuronal responses show a decrease (Newsome et al., 1988). Then, extraretinal signals carried in the dorsal-medial part of MST (MSTd) are thought to take over to maintain pursuit eye velocity (Newsome et al., 1988; Ilg and Thier, 2003; Ono and Mustari, 2012). It is known that this visuomotor processing underlying the initiation of smooth pursuit is variable, which could depend on behavioral states or experience of eye movements (Tabata et al., 2006; Barnes, 2008). Previous studies have demonstrated that an adaptive change of initial pursuit is induced by a step-ramp tracking with two different velocities (double-step paradigm) in human (Fukushima et al., 1996; Ogawa and Fujita, 1997) and monkeys (Kahlon and Lisberger, 1996; Nagao and Kitazawa, 1998; Takagi et al., 2000; Ono and Mustari, 2012). Typically, 100 to 200 sequential trials are used for a double-step paradigm, which alter the gain of visuomotor transmission in pursuit pathways.

There is another form of visuomotor behavior associated with the gain modulation during smooth pursuit. Previous studies have shown that a brief perturbation of visual target motion induces a corresponding perturbation of eye motion in humans and monkeys (Schwartz and Lisberger, 1994; Churchland and Lisberger, 2002, 2005; Nuding et al., 2008; Ono et al., 2010). The advantage of using the perturbation response is to measure the gain of visuomotor transmission during ongoing smooth pursuit at different speeds. Typically, a single cycle of sinusoidal motion is introduced during steady-state pursuit. The perturbation responses of eye motion are dependent on ongoing pursuit velocity, even though the perturbation frequency and amplitude are constant. This nonlinear perturbation response (gain slope) reveals a dynamic gain control mechanism in pursuit. Evidence for dynamic gain control was first proposed by Robinson (1965) as spontaneous oscillations occurred during smooth pursuit, but not during fixation of a stationary target. Schwartz and Lisberger (1994) have also shown that during fixation of a stationary target, perturbed eye responses are smaller compared with those during pursuit.

Although both pursuit adaptation and dynamic gain control require a change in the visuomotor gain, it has not been fully understood whether these visuomotor processing are supported by a common neuronal mechanism. Therefore, this study was designed to compare the perturbation responses modulated by adaptation and dynamic gain control. First, a brief perturbation of target motion was applied during step-ramp tracking at different velocities to verify the dynamic regulation of the visuomotor gain. Second, the perturbation responses were tested pre- and post-adaptation to define how smooth pursuit adaptation affects perturbed eye velocity. The dynamic gain control led to changes in the perturbation response bidirectionally, but here we found that pursuit adaptation had a significant effect on the perturbation response that was specific to the adapted direction. This is consistent with directional specificity of adaptive changes in pursuit initiation. These results suggest that there might be different underlying mechanisms responsible for pursuit adaptation and dynamic gain control.

## Materials and methods

### Surgical procedures

A detailed description of our surgical procedures can be found in earlier publications (e.g., Ono and Mustari, 2009, 2012). Surgical procedures, carried out under aseptic conditions using isoflurane anesthesia (1.25–2.5%), were used to stereotaxically implant a titanium head stabilization post (Crist Instruments, MD). In the same surgery, a scleral search coil for measuring eye movements (Fuchs and Robinson, 1966) was implanted underneath the conjunctiva of one eye (Judge et al., 1980). All surgical procedures were performed in strict compliance with National Institutes of Health Guide for the Care and Use of Laboratory Animals and the protocols were reviewed and approved by the Institutional Animal Care and Use Committee (IACUC) at the University of Washington.

### Behavioral paradigms

Behavioral data were collected from two normal juvenile rhesus monkeys (*Macaca mulatta*), weighing 5–8 kg in this study. During all experiments, monkeys were seated in a primate chair (Crist Instruments, MD) with their head stabilized in the horizontal stereotaxic plane. Visual stimuli were rear projected on a tangent screen 57 cm distant. All of our monkeys were extensively trained to perform a fixation task and track a small diameter (0.2°) target spot moving in sinusoidal or step-ramp trajectories. Eye position signals (see below) were calibrated by requiring the monkey to fixate a small target spot at known horizontal and vertical eccentricities. The monkeys were rewarded with juice for fixating the target with the eyes (within a ± 3° window for duration of 0.5 s). Motion of the target spot was produced by a computer controlled two-axis mirror galvanometer setup (General Scanning, Watertown, MA).

Adaptive changes of initial smooth pursuit were produced by a step-ramp tracking with two different velocities (double-step paradigm). In the adaptation paradigm, the monkey tracked double-steps of target speed that step-up (10°/s to 30°/s) or step-down (20°/s to 5°/s). In the step-up paradigm, the target begins moving at 10°/s for the first 100 ms and then changes to 30°/s for the remainder of the trial. In the step-down paradigm, the target begins moving at 20°/s for first 100 ms and then changes to 5°/s for the remainder of the trial. Smooth pursuit adaptation was evaluated during > 150 sequential trials for each adaptation paradigm (Figures 2, 6). For each adaptation session, one direction (left or rightward) was chosen randomly for adaptation (double-step paradigm), whereas the opposite direction served as a control (normal step-ramp paradigm) direction.

**Figure 1:**
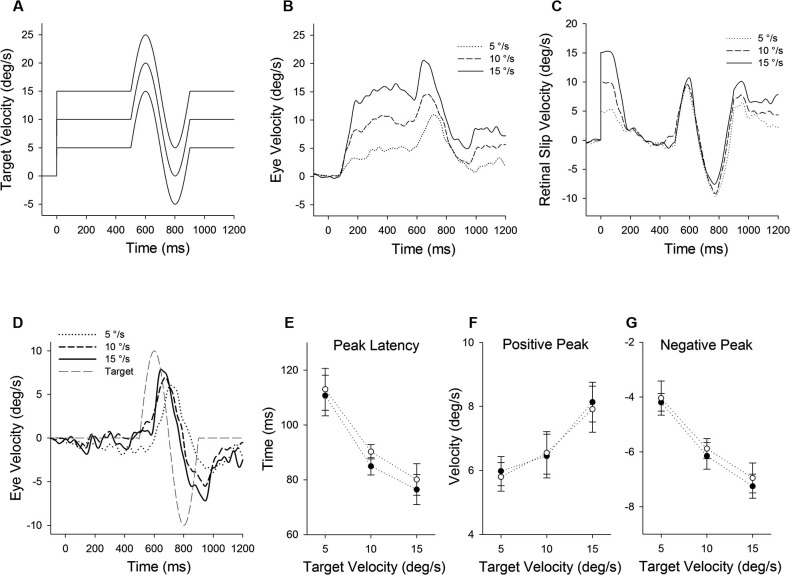
**Eye motion evoked by a brief perturbation of target motion during smooth pursuit**. **(A)** A single cycle of sinusoidal motion (positive-first, 2.5 Hz, ± 10°/s) is introduced (500 ms after target onset) during step-ramp tracking at different target velocities (5, 10 and 15°/s). **(B)** Mean eye velocity traces at three different target velocities are shown as a function of time. **(C)** Mean retinal slip velocity traces are shown as a function of time. **(D)** Perturbed eye velocity traces calculated as the difference between eye velocity during step-ramp tracking with and without the perturbation at different ramp velocities (5, 10 and 15°/s). **(E)** Mean and standard deviation (SD) values of perturbation latency, **(F)** positive peak velocity and **(G)** negative peak velocity are shown as a function of target velocity for two monkeys. Open and filled symbols indicate monkey-P and B, respectively.

Target perturbation using a single cycle of sinusoidal motion (2.5 Hz, ± 10°/s) with first half-cycle increasing the stimulus velocity (positive-first; Figure 1A) or first half-cycle decreasing the velocity (negative-first; Figure 4A) was introduced during steady-state pursuit phase (500 ms after target onset) at different target velocities (5, 10 and 15°/s). The perturbation trials were tested before and after adaptation paradigms. Trials with three target velocities (5, 10 and 15°/s) and directions of target motion (left or rightward) were randomized. Each target velocity in left or rightward direction was repeated at least 15 times. We conducted one set of adaptation trials and perturbation testing (pre- and post-adaptation) in a given experimental session. Therefore, 30 experimental sessions in total were conducted on different days, which include 20 step-up and 10 step-down experiments for leftward or rightward pursuit directions in two monkeys.

### Data Collection and analysis

Eye movements were detected using electromagnetic methods and scleral search coil systems (CNC Electronics, Seattle, WA). Eye and target position feedback signals were processed with anti-aliasing filters at 200 Hz using 6-pole Bessel filters prior to digitization at 1 kHz with 16-bit precision using CED-Power1401 hardware (Cambridge Electronic Designs, Cambridge, England). Eye velocity was generated by digital differentiation of the position arrays using a central difference algorithm in Matlab (Mathworks, Natick, MA). Pursuit initiation during step-ramp tracking was taken as the time that average eye speed reached ≥ 3 SD above the pre-trial values during fixation. To quantitatively estimate smooth pursuit adaptation, initial acceleration was calculated as the average eye acceleration in the first 100 ms period of pursuit. The perturbation response of eye velocity was determined by the difference of the maximum to the subsequent minimum of eye velocity following a perturbation (Figure 1B). Furthermore, the relative perturbation response is calculated by subtracting the steady-state pursuit velocity during control testing without the perturbation from the eye velocity with the perturbation, which allow us to estimate the positive and negative peak velocities at different ramp speeds (5, 10 and 15°/s) (Figure 1D) and pre- and post-adaptation (Figure 3). The perturbation response latency, which indicates the delay of the ocular response to target perturbation, was determined by the first peak eye velocity induced by sinusoidal target motion. The analysis of covariance (ANCOVA) was used to compare two regression lines by testing the effect of categorical factor (pre- and post-adaptation) on a dependent variable (perturbation response) while controlling for the effect of a continuous co-variable (target velocity). Regression lines were compared by studying the interaction of the categorical variable with the continuous independent variable (Figure 8). All statistical tests including were executed with an alpha level of 0.05.

**Figure 2:**
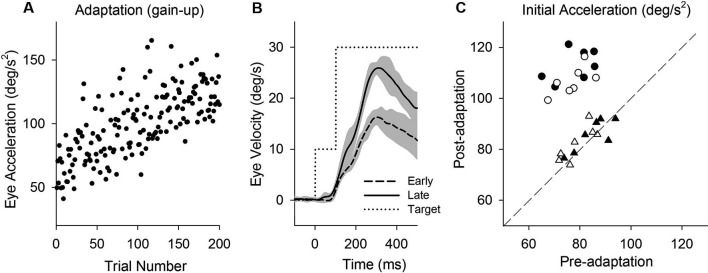
**Smooth pursuit adaptation during the step-up (gain-increase) paradigm**. **(A)** Initial eye acceleration in the first 100 ms of tracking during adaptation plotted as a function of trial number. **(B)** Mean eye velocity traces are shown for early and late in adaptation. During adaptation, the target began moving at 10°/s for the first 100 ms and stepped up to 30°/s (dotted line). **(C)** Mean eye acceleration values (first 100 ms) show significant adaptive changes in the direction of a step-up paradigm (circle symbols) but not in the control direction (triangle symbols). Open and filled symbols indicate monkey-P and B, respectively.

**Figure 3:**
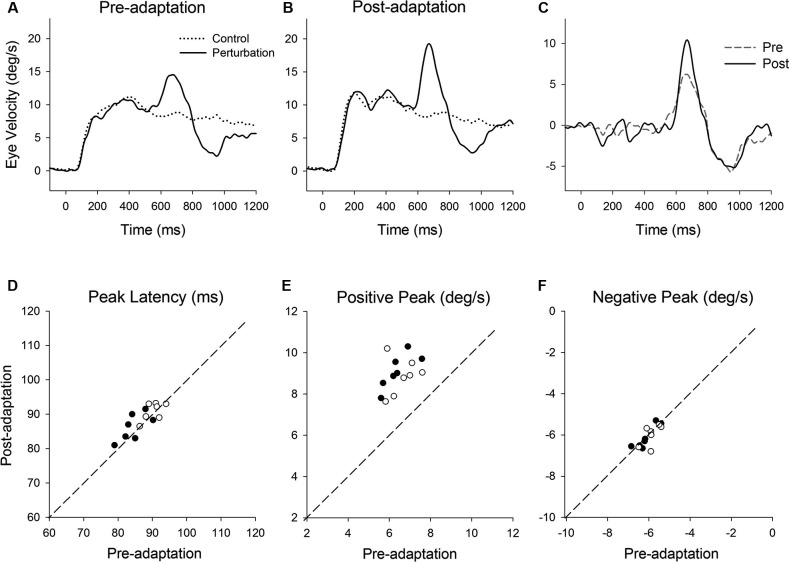
**(A)** Representative eye velocity traces during step-ramp tracking with (solid lines) and without (dotted lines) the perturbation are shown pre- and **(B)** post-adaptation. **(C)** Perturbation responses obtained by subtracting the eye velocity during control testing from the perturbed eye velocity pre- (broken line) and post-adaptation (solid line). **(D–F)** Summaries of the perturbation responses for 14 experiments of gain-increase adaptation for two monkeys. **(D)** Mean values of perturbation latency, **(E)** positive peak velocity and **(F)** negative peak velocity are shown pre- and post-adaptation. Open and filled symbols indicate monkey-P and B, respectively.

## Results

### Perturbation responses during smooth pursuit

Figure 1 illustrates representative responses to a target perturbation (positive-first; Figure 1A) during ongoing pursuit at different target speeds (5, 10 and 15°/s), while the perturbation frequency and amplitude are constant. Mean eye velocity traces during step-ramp tracking with perturbations are shown in Figure 1B. Perturbed eye velocity is estimated by the difference of the maximum to the subsequent minimum of eye velocity after target perturbation. Eye velocity traces document that the perturbation responses were enhanced when target speed is higher, as described previously (Schwartz and Lisberger, 1994; Churchland and Lisberger, 2002; Ono et al., 2010). For example, mean values of perturbed eye velocity increased significantly from 9.9 ± 1.4°/s (mean ± standard deviation) at 5°/s target speed to 15.7 ± 1.8°/s at 15°/s target speed (*F*_2, 27_ = 36.2, *P* < 0.001, one-way ANOVA). Furthermore, peak eye acceleration induced by the perturbation increased significantly from 87.7 ± 31.0°/s at 5°/s target speed to 219.2 ± 57.1°/s at 15°/s target speed (*F*_2, 27_ = 23.9, *P* < 0.001, one-way ANOVA). This was the case even though retinal slip velocity induced by the target perturbation showed no significant change in three different target velocities (Figure 1C) (18.9 ± 0.84°/s at 5°/s target speed to 18.4 ± 1.25°/s at 15°/s target speed; *F*_2, 27_ = 0.97, *P* = 0.39, one-way ANOVA). Perturbed retinal slip velocity is estimated by the difference of the maximum to the subsequent minimum of retinal slip velocity during target perturbation.

Eye velocity traces in Figure 1D show the perturbation responses obtained by subtracting the eye velocity during control testing (without perturbation) from the perturbed eye velocity. The relative eye velocity traces allow us to estimate the positive and negative perturbation responses at different ramp speeds (5, 10 and 15°/s). Figures 1E–G show mean values of perturbation responses in two monkeys. The latencies of positive peak responses monotonically decreased with increasing ramp velocities (*F*_2, 39_ = 140, *P* < 0.001, one-way ANOVA) (Figure 1E). Furthermore, increasing ramp velocities led to a significant increase in positive peak velocity (*F*_2, 39_ = 51.0, *P* < 0.001, one-way ANOVA) (Figure 1F), while negative peak velocity decreased with increasing ramp velocities (*F*_2, 39_ = 188, *P* < 0.001, one-way ANOVA) (Figure 1G). Both latency and magnitude reflect dynamic gain control (Schwartz and Lisberger, 1994; Churchland and Lisberger, 2002, 2005; Ono et al., 2010).

### Perturbation responses (positive-first) pre- and post-adaptation (gain-increase)

This study attempts to determine the effects of smooth pursuit adaptation on the perturbation responses. Figure 2 illustrates smooth pursuit adaptation using the gain-increase (step-up) paradigm (10–30°/s). Trial-by-trial eye acceleration data across trials are plotted in Figure 2A. The time course of the adaptation showed increases gradually until 200 trials. Mean eye velocity traces in early (first 10 of 200 trials) and late trials (last 10 of 200 trials) during the step-up paradigm are shown in Figure 2B. To provide an estimate of adaptation, initial acceleration was calculated as the average eye acceleration in the first 100 ms period of pursuit. Initial eye acceleration showed a significant increase in late trials compared with that in early trials (61.0 ± 13.4°/s^2^, 1st 10 of 200 trials; 128.7 ± 12.8°/s^2^, last 10 of 200 trials; *T*_18_ = 11.7, *P* < 0.001, unpaired *t*-test). Figure 2C shows mean values of initial eye acceleration during control trials using normal step-ramp paradigm (ramp velocity of 10°/s) in rightward and leftward directions. Initial eye acceleration in the adapted direction (circle symbols) significantly increased in post-adaptation (79.5 ± 6.9°/s^2^, preadapt; 109.9 ± 6.6°/s^2^, postadapt; *T*_13_ = 18.2, *P* < 0.001, paired *t*-test), whereas the control direction (triangle symbols) showed no significant change in initial eye acceleration (81.2 ± 7.1°/s^2^, preadapt; 82.9 ± 6.5°/s^2^, postadapt; *T*_13_ = 1.8, *P* = 0.10, paired *t*-test).

Figure 3 shows representative perturbation responses during ramp velocity of 10°/s pre- and post-adaptation. Mean eye velocity traces during step-ramp tracking with and without the perturbation are shown (Figures 3A, B). Figure 3C shows the perturbation responses obtained by subtracting the pursuit eye velocity during control testing (without perturbation) from the perturbed eye velocity pre- and post-adaptation. The results of 14 experiments of gain-increase adaptation in two monkeys are shown in Figures 3D–F. These perturbation responses were tested during step-ramp tracking of 10°/s. The latencies of positive peak responses remained constant following adaptation (87.6 ± 3.9 ms, pre; 88.9 ± 3.4 ms, post; *T*_13_ = 1.94, *P* = 0.11, paired *t*-test for the whole group). This is the case even though positive peak velocity showed significant increases in post-adaptation compared with values of preadapted trials (6.5 ± 0.66°/s, pre; 9.0 ± 0.83°/s, post; *T*_13_ = 11.9, *P* < 0.001, paired *t*-test for the whole group). In contrast, negative peak velocity showed no significant change in post-adaptation (−6.0 ± 0.43°/s, pre; −6.1 ± 0.51°/s, post; *T*_13_ = 0.65, *P* = 0.53, paired *t*-test for the whole group).

### Perturbation responses (negative-first) pre- and post-adaptation (gain-increase)

**Figure 4:**
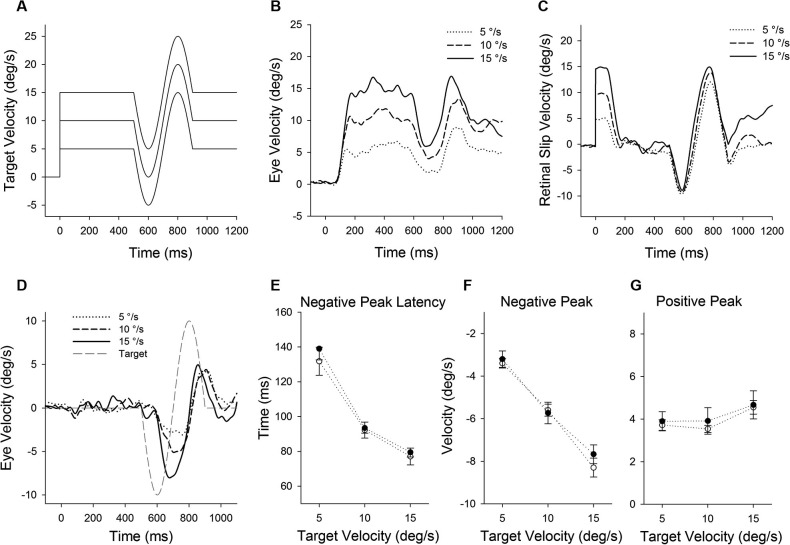
**(A)** A single cycle of sinusoidal motion (negative-first, 2.5 Hz, ± 10°/s) is introduced (500 ms after target onset) during step-ramp tracking at different target velocities (5, 10 and 15°/s). **(B)** Mean eye velocity traces at three different target velocities are shown as a function of time. **(C)** Mean retinal slip velocity traces are shown as a function of time. **(D)** Perturbed eye velocity traces calculated as the difference between eye velocity with and without the perturbation at different ramp velocities (5, 10 and 15°/s). **(E)** Mean and standard deviation (SD) values of negative peak latency, **(F)** modulation of negative peak and **(G)** positive peak are shown as a function of target velocity. Open and filled symbols indicate monkey-P and B, respectively.

Figure 4 illustrates responses to a target perturbation (negative-first; Figure 4A) during ongoing pursuit at different target speeds (5, 10 and 15°/s). Mean eye velocity traces document that the perturbation responses are enhanced when target speed is higher (Figure 4B). This was the case even though retinal slip velocity induced by the target perturbation showed no significant change in three different target velocities (Figure 4C) (*F*_2, 27_ = 1.66, *P* = 0.21, one-way ANOVA). Eye velocity traces in Figure 4D show the difference between the responses with and without the perturbation at different ramp speed (5, 10 and 15°/s). Figures 4E–G show mean values of perturbation responses in two monkeys. The latencies of negative peak responses monotonically decreased with increasing ramp velocities (*F*_2, 15_ = 157, *P* < 0.001, one-way ANOVA) (Figure 4E). Increasing ramp velocities led to a significant decrease in negative peak velocity (*F*_2, 15_ = 192, *P* < 0.001, one-way ANOVA) (Figure 4F), whereas there was no significant change in positive peak velocity (*F*_2, 15_ = 2.27, *P* = 0.14, one-way ANOVA) (Figure 4G).

**Figure 5:**
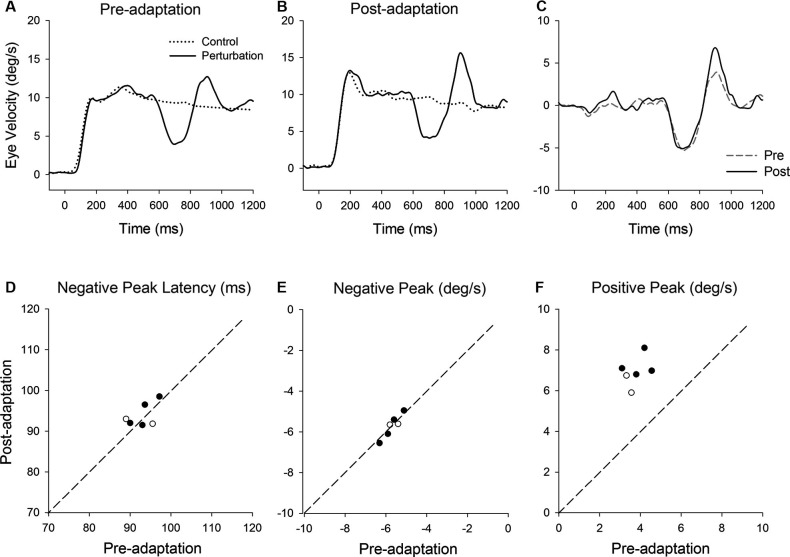
**(A)** Mean eye velocity traces during step-ramp tracking with (solid lines) and without (dotted lines) the perturbation are shown pre- and **(B)** post-adaptation. **(C)** The difference between two eye velocity traces pre- (broken line) and post-adaptation (solid line). **(D)** Mean values of perturbation latency, **(E)** negative peak velocity and **(F)** positive peak velocity are shown pre- and post-adaptation. Open and filled symbols indicate monkey-P and B, respectively.

Figure 5 shows representative perturbation responses during ramp velocity of 10°/s pre- and post-adaptation. Mean eye velocity traces during step-ramp tracking with and without the perturbation (negative-first) are shown (Figures 5A, B). Figure 5C shows the perturbation responses obtained by subtracting the eye velocity during control testing (without perturbation) from the perturbed eye velocity pre- and post-adaptation. The latencies of negative (first) peak responses did not show significant changes in post-adaptation (93.0 ± 3.1 ms, pre; 93.9 ± 2.9 ms, post; *T*_5_ = 0.71, *P* = 0.51, paired *t*-test for the whole group) (Figure 5D). The negative (first) peak velocity also remained constant following adaptation (−5.68 ± 0.42°/s, pre; −5.71 ± 0.55°/s, post; *T*_5_ = 0.27, *P* = 0.79, paired *t*-test for the whole group) (Figure 5E). In contrast, pursuit adaptation yielded larger responses in positive peak velocity compared with preadapted trials (3.8 ± 0.55°/s, pre; 6.9 ± 0.71°/s, post; *T*_5_ = 10.9, *P* < 0.001, paired *t*-test for the whole group) (Figure 5F).

### Perturbation responses (positive-first) pre- and post-adaptation (gain-decrease)

**Figure 6:**
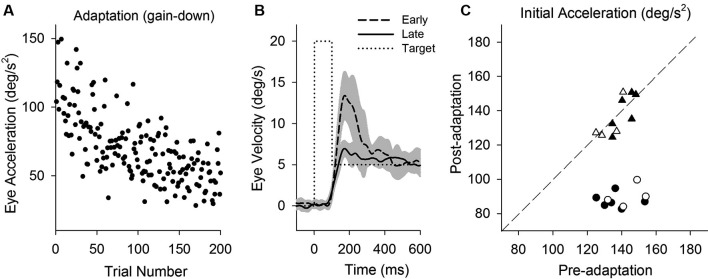
**Smooth pursuit adaptation during the step-down (gain-decrease) paradigm**. **(A)** Initial eye acceleration in the first 100 ms of tracking plotted as a function of trial number. **(B)** Mean eye velocity traces are shown for early and late in adaptation. During adaptation, the target began moving at 20°/s for the first 100 ms and stepped down to 5°/s (dotted line). **(C)** Mean eye acceleration values (first 100 ms) show significant adaptive changes in the direction of a step-down paradigm (circle symbols) but not in the control direction (triangle symbols). Open and filled symbols indicate monkey-P and B, respectively.

Smooth pursuit adaptation using a step-down paradigm (20–5°/s) is designed to decrease initial pursuit acceleration during step-ramp tracking. Initial eye acceleration values for individual trials during adaptation are plotted in Figure 6A, showing a decrease in initial pursuit gain gradually across 200 trials. Mean eye velocity traces in early and late trials are shown in Figure 6B. For example, initial eye acceleration (the first 100 ms of eye motion) decreased significantly in late trials compared with early trials (115.8 ± 32.0°/s^2^, first 10 of 200 trials; 66.1 ± 22.5°/s^2^, last 10 of 200 trials; *T*_18_ = 11.7, *P* < 0.001, unpaired *t*-test). Figure 6C shows mean values of initial eye acceleration during control trials using normal step-ramp paradigm (ramp velocity of 15°/s) in rightward and leftward directions. Initial eye acceleration in the adapted direction (circle symbols) significantly decreased in post-adaptation (138.5 ± 9.9°/s^2^, preadapt; 88.6 ± 5.2°/s^2^, postadapt; *T*_9_ = 16.1, *P* < 0.001, paired *t*-test), whereas the control direction (triangle symbols) showed no significant change in initial eye acceleration (137.1 ± 6.7°/s^2^, preadapt; 136.0 ± 9.6°/s^2^, postadapt; *T*_9_ = 0.49, *P* = 0.63, paired *t*-test).

**Figure 7:**
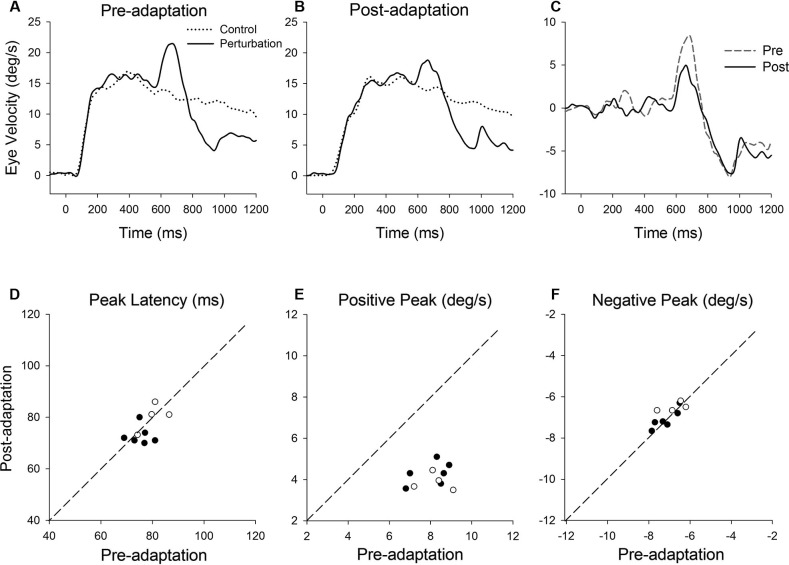
**(A)** Mean eye velocity traces during step-ramp tracking with (solid lines) and without (dotted lines) the perturbation are shown pre- and **(B)** post-adaptation. **(C)** The difference between two eye velocity traces pre- (broken line) and post-adaptation (solid line). **(D)** Mean values of perturbation latency, **(E)** positive peak velocity and **(F)** negative peak velocity are shown pre- and post-adaptation. Open and filled symbols indicate monkey-P and B, respectively.

Figure 7 shows representative perturbation responses during ramp velocity of 15°/s pre- and post-adaptation. Mean eye velocity traces during step-ramp tracking with and without the perturbation are shown pre- and post-adaptation of gain-decrease (Figures 7A, B). Figure 7C shows subtraction of the two eye velocity traces. The results of 10 experiments of gain-decrease adaptation in two monkeys are shown in Figures 7D–F. These perturbation responses were tested during step-ramp tracking of 15°/s pre- and post-adaptation. The latencies of positive peak responses remained constant post-adaptation (77.3 ± 4.9 ms, pre; 75.9 ± 5.6 ms, post; *T*_9_ = 0.88, *P* = 0.40, paired *t*-test for the whole group). This was the case even though the positive peak velocity decreased significantly in post-adaptation compared with preadapted trials (8.1 ± 0.81°/s, pre; 4.1 ± 0.53°/s, post; *T*_9_ = 14.6, *P* < 0.001, paired *t*-test for the whole group). In contrast, negative peak velocity showed no significant change in post-adaptation (−7.0 ± 0.58°/s, pre; −6.9 ± 0.48°/s, post; *T*_9_ = 1.38, *P* = 0.20, paired *t*-test for the whole group).

### Effects of adaptation on perturbation responses at different pursuit velocities

**Figure 8:**
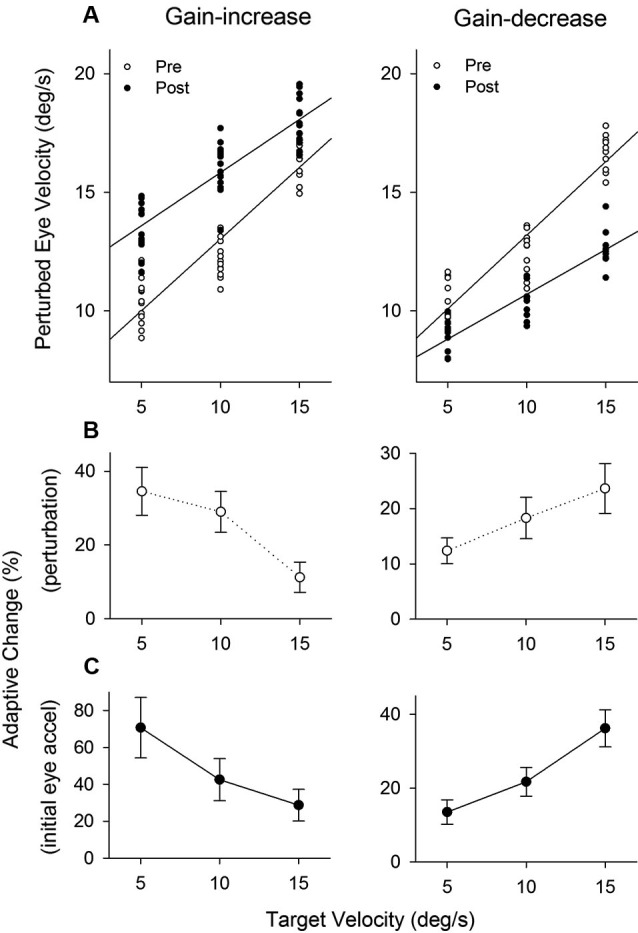
**The effect of pursuit adaptation on dynamic gain control**. **(A)** Mean eye velocity of all the perturbation responses at three different target velocity conditions (5, 10 and 15°/s) for gain-increase (left) and decrease (right) experiments (top panels). Open and filled circles indicate pre- and post-adaptation, respectively. Straight lines are linear regression fits between perturbed eye velocity and target velocity. Two regression lines of pre- and post-adaptation have different slopes in gain-increase and decrease adaptation. **(B–C)** Percentages of adaptive change in the perturbation response and initial eye acceleration (first 100 ms of tracking) at three different target velocities for gain-increase and decrease adaptation.

Figure 8A shows the population of perturbation responses tested at three different target velocity conditions (5, 10 and 15°/s) pre- and post-adaptation. Linear regression fits are shown in gain-increase adaptation pre (*r*^2^ = 0.86, slope = 0.60, *P* < 0.001) and post trials (*r*^2^ = 0.75, slope = 0.45, *P* < 0.001), and gain-decrease adaptation pre (*r*^2^ = 0.87, slope = 0.62, *P* < 0.001) and post trials (*r*^2^ = 0.81, slope = 0.38, *P* < 0.001). To compare two regression lines pre and post trials, we used an analysis of covariance (ANCOVA) (see Section Methods). The results showed that the interaction of the adapted state (pre- and post-testing) and tracking velocity was significantly different (*F*_1, 56_ = 7.68, *P* < 0.01), indicating that two regression lines (pre and post) in gain-increase adaptation have different slopes. Similarly, for gain-decrease adaptation, the slopes of two regression lines for pre- and post-adaptation were significantly different (*F*_1, 78_ = 19.12, *P* < 0.001). Figure 8B shows relative changes of the perturbation response in each target velocity, defined as the percentage difference between pre and post adaptation trials. The percentage change of perturbed eye velocity significantly decreased with higher target velocities for gain-increase adaptation (*F*_2, 39_ = 69.24, *P* < 0.001, one-way ANOVA) and increased with higher velocities for gain-decrease adaptation (*F*_2, 27_ = 24.05, *P* < 0.001, one-way ANOVA).

Furthermore, percentages of adaptive change in initial eye acceleration (first 100 ms of step-ramp tracking) at three different target velocities are shown in Figure 8C. For the gain-increase paradigm, adaptation yielded larger percentage changes in initial eye acceleration at lower target speeds (*F*_2, 39_ = 40.7, *P* < 0.001, one-way ANOVA). In contrast, the gain-decrease adaptation caused smaller percentage changes at lower speeds (*F*_2, 27_ = 77.5, *P* < 0.001, one-way ANOVA).

## Discussion

The present study characterized the properties of the gain modulation of visuomotor transmission associated with smooth pursuit adaptation and dynamic gain control. A brief perturbation of target motion was applied during step-ramp tracking pre- and post-adaptation using a double-step paradigm. The results showed that the magnitude of the perturbation response was modulated by pursuit adaptation.

### The effects of smooth pursuit adaptation on the perturbation responses

It has been demonstrated that a short-duration single cycle of sinusoidal motion (motion perturbation) induces a corresponding response of eye motion. When a perturbation of target motion is applied during smooth pursuit at different target velocities, perturbed eye velocity increases as a function of baseline pursuit velocity (Schwartz and Lisberger, 1994; Churchland and Lisberger, 2002, 2005; Nuding et al., 2008; Ono et al., 2010). This nonlinear response (gain slope) is thought to be based on a dynamic (on-line) gain control mechanism in smooth pursuit. The dynamic gain control is known to regulate an internal gain parameter in pursuit, where higher target velocities yield higher gains in both increasing (toward the pursuit direction) and decreasing (the opposite direction) perturbation responses. Previous studies have revealed that micro-electrical stimulation in the frontal eye field (FEF) enhanced the eye motion evoked by a target perturbation, regardless of the direction of ongoing pursuit (Tanaka and Lisberger, 2001, 2002). Therefore, these authors suggested that the FEF plays a role in setting the internal gain of smooth pursuit.

The results in this study showed that smooth pursuit adaptation had a significant effect on the perturbation response in the adapted direction. In contrast, the perturbation response in the control (opposite) direction showed no significant change in post-adaptation. These results are not consistent with dynamic gain control, which showed a biphasic change in perturbed eye velocity (Churchland and Lisberger, 2002; Ono et al., 2010). Schwartz and Lisberger (1994) have shown that the perturbation response is direction selective only if the target perturbation is orthogonal to the axis of ongoing pursuit. It is known that the pursuit adaptation induced by a double-step paradigm shows directional specificity, where adaptive changes occur in the direction of the double-step paradigm but not in the other (control) direction. These results indicate that the change in the perturbation response following adaptation is direction selective. Therefore, the gain modulation associated with pursuit adaptation and dynamic gain control could be supported by different visuomotor processing. This is also supported by the results from the negative-first perturbation paradigm shown in Figures 4, 5. For the negative-first paradigm, the positive peak velocity (toward the pursuit direction) did not show the nonlinear response (gain slope) at different target velocities (Figure 4), as described previously (Churchland and Lisberger, 2002). If dynamic gain control and pursuit adaptation are supported by a common visuomotor mechanism, we would expect no change in positive gain even after pursuit adaptation. However, the positive peak velocity showed significant increases in post-adaptation, which was specific to the adapted direction (Figure 5). These results support the suggestion that there might be different underlying mechanisms between adaptation and dynamic gain control.

Furthermore, the perturbation responses were tested at three different target velocity conditions in post-adaptation. Figure 8A showed that smooth pursuit adaptation yielded not only a modulation in the gain of the perturbation response, but also a change of the gain slope at different target velocities. These results indicate that pursuit adaptation alters the efficacy of dynamic gain control. The effect of adaptation on the perturbation response seems to be based on the adaptive change. Thus, the change of the gain slope for gain-increase and decrease adaptation could be predicted by the percentage change of the perturbation response. Here it is important to note that the nonlinear perturbation response (gain slope) at different target velocities is induced by constant retinal slip velocity (see Figure 1C), whereas adaptive changes in initial pursuit gain are dependent on different retinal slip velocities. Taken together, these results support the suggestion that percentage changes of pursuit adaptation at different target velocities could influence the dynamic gain control mechanism.

### Possible neuronal pathways involving adaptation of perturbation responses

Even though both pursuit adaptation and dynamic gain control require a change in the gain of visuomotor transmission, it seems that they are supported, at least in part, by different underlying mechanisms. Although neuronal mechanisms and regions involved in dynamic gain control are incompletely understood, it has been suggested that cortical pursuit areas including FEF are involved in pursuit gain regulation (Tanaka and Lisberger, 2001, 2002; Nuding et al., 2008, 2009; Ono et al., 2010). In contrast, previous studies provided strong evidence that the direction selective adaptation of smooth pursuit is attributed to plasticity mechanisms in the cerebellum, including the floccular complex and oculomotor vermis (Kahlon and Lisberger, 2000; Nagao and Kitazawa, 2000; Takagi et al., 2000). However, it has not been determined whether cerebellar plasticity mechanisms could influence cortical visuomotor signals. Tanaka and Lisberger (2001, 2002) have demonstrated that micro-electrical stimulation in the FEF enhanced the eye motion evoked by a target perturbation, suggesting that the FEF plays a role in dynamic gain control which regulates the visuomotor output even facing same stimulus velocity. Furthermore, a recent study using transcranial magnetic stimulation (TMS) showed that disrupting neuronal activity in the FEF attenuated the efficacy of dynamic gain control for pursuit (Nuding et al., 2009). The FEF is known to have reciprocal connections with extrastriate visual motion areas including MT and MST (Huerta et al., 1987; Tian and Lynch, 1996). The cortical visual motion pathways are responsible for beginning the process of converting visual motion information into commands for eye motion (Krauzlis, 2004). The cortical visual motion signals must be processed further in the oculomotor regions of the cerebellum and vestibular nuclei. It has been also demonstrated that the FEF receives feedback signals from the cerebellum through the oculomotor thalamus (Huerta et al., 1987; Lynch et al., 1994; Tian and Lynch, 1997). Previous studies have shown that the FEF and oculomotor thalamus contribute to pursuit initiation which is thought to be an open-loop response for visuomotor control (Gottlieb et al., 1994; Fukushima, 2003; Tanaka, 2005; Ono and Mustari, 2009; Mahaffy and Krauzlis, 2011). The source of visual and oculomotor signals in the oculomotor thalamus includes the deep cerebellar and the vestibular nuclei (Lang et al., 1979; Kalil, 1981; Asanuma et al., 1983). Therefore, it is possible that feedback signals from the cerebellum to the thalamocortical pathway may play a role in regulating the visuomotor gain in the cortical pursuit system.

Taken together, the characteristic changes in the gain of visuomotor transmission associated with pursuit adaptation and dynamic gain control could be explained by the differential neuronal mechanisms. Furthermore, this study provides evidence that two different visuomotor mechanisms could interact with each other to regulate pursuit gain. Although the cortical pursuit system involved in pursuit adaptation is uncertain, the cortical visuomotor processing may contribute differently to pursuit adaptation and dynamic gain control in the cortico-cerebellar pathways.

## Conflict of interest statement

The author declares that the research was conducted in the absence of any commercial or financial relationships that could be construed as a potential conflict of interest.
